# Fellowships, Grants, & Awards

**Published:** 2006-06

## *In Utero* Exposure to Bioactive Food Components and Mammary Cancer Risk (R21)

*In utero* is a vulnerable period. *In utero* exposures are important determinants of some cancers occurring in children and young adults. For example, exposure to ionizing radiation *in utero* promotes childhood leukemia, and maternal use of diethylstilbestrol during pregnancy has been linked to clear-cell adenocarcinoma of the vagina in their daughters. In addition, maternal diets, specifically the consumption of vegetables, fruits, and protein, are linked to decreased risk of childhood leukemia.

The prenatal period is critical in the development of the mammary gland. During this time, the mammary gland is in a largely undifferentiated state, making it particularly vulnerable to a host of environmental forces. Inappropriate nutritional status or exposure to environmental chemicals and the accompanied alteration in growth and endocrine homeostasis may permanently change the fetus structure, physiology, and metabolism, thereby predisposing it to various diseases in later life, including mammary cancer.

In utero *exposures and human breast cancer risk*. Epidemiological studies suggest that altering the intrauterine nutritional status can increase mammary cancer risk. Failure of the materno–placental supply line to satisfy fetal nutrient requirements can result in a range of fetal adaptations and developmental changes. Birth weight is a gross surrogate marker for shifts in a host of metabolic processes. Many, but not all, studies reveal a positive relationship between increased birth weight and breast cancer risk. Likewise, other indicators of fetal size such as increased placental weight and birth length are positively correlated with breast cancer risk in the offspring. Recent studies suggest that birth weight is independent from neonatal growth patterns and the timing of puberty as a risk factor for breast cancer.

In addition to nutrition, the hormonal environment in the womb may play an important role in programming lifelong risk for breast cancer in female offspring. A reduction in circulating levels of estrogens and insulin-like growth factor-1 (IGF-1), and/or elevated levels of progesterone, androgens, human chorionic gonadotrophin, IGF-1 binding proteins 1 and 3, cortisol, and insulin have been associated with reduced risk. Such hormonal and growth factor changes are observed during preeclampsia. Maternal preeclampsia has been associated with a reduction in the female offspring's later risk for breast cancer after adjustment for a variety of potential confounders.

### Mammary gland development, dietary modification, and breast cancer risk

Proliferation of primitive ductal structures in the newborn breast leads to branching and terminal end buds (TEBs). The expansion of TEBs represents an opportunity for malignant transformation because they contain pluripotent mammary stem cells. In fact, *in utero* exposures that bring about an increase in TEBs coincide with increased mammary carcinogenesis. Evidence exists that providing maternal diets that contain elevated amounts of n-6 polyunsaturated fatty acids (PUFAs) and genistein not only increased TEBs, but also reduced the differentiation of TEBs to lobuloalveolar units. These diets also increased subsequent chemically induced mammary cancer in the offspring. In addition, prenatal exposure to environmental agents, such as bisphenol A or dioxin, results in alteration in the development of the mammary gland that may predispose to the development of cancers later in life. Some of this response may relate to changes in hormonal and growth factor status, including estrogen and IGF-1.

### Estrogen, dietary modification, and breast cancer risk

Greater estrogen exposure throughout a woman’s life has been identified as a major risk factor for the development of breast cancer. *In utero* exposures to the mammary gland can achieve concentrations 10–100 times the estrogen levels occurring later in life. Dietary factors, such as genistein and fat, which influence estrogen exposure to the fetus, are related to subsequent cancer risk in several model systems. However, the response may not be totally explained by estradiol, since n-3 fatty acid– rich diets fed to pregnant rats elevate this hormone, but reduce mammary cancer incidence in the offspring.

It is possible that intrauterine exposure to other hormones, environmental hormone mimics, or antagonists may also affect breast cancer susceptibility. Androgen exposure *in utero* may confer long-term protection against breast cancer by antagonizing the effects of estrogens on fetal breast ductal development. Dietary fatty acids, phytoestrogens, alcohol, and lycopene are among the various bioactive food components reported to influence androgen concentrations. Environmental agents with estrogenic agonist or antagonist activity may also alter gene expression during development that may lead to functional deficits later in life that predispose to cancer development. Thus, there is the need for future studies focusing on uncovering the mechanisms responsible for the protective and detrimental effects of exposure to bioactive food components and other environmental agents *in utero* on breast cancer risk. These studies should attempt to address more comprehensively the changes in all potentially relevant pregnancy hormones and growth factors.

### IGF, dietary modification, and breast cancer risk

The IGF-1 system may play a crucial role in the increased risk that heavier newborns have of developing breast cancer later in life. Birth weight is positively associated with increased insulin and IGF-1 concentrations. Analysis of mammary gland development in knockout mice made deficient in IGF-I or in the IGF-I receptor demonstrates the importance of the IGF system for normal mammary gland development because these mice have diminished TEB development. Observational and preclinical studies provided added evidence that one or more components of the IGF-1 system may be intimately linked to the process of carcinogenesis in the mammary gland. Transgenic mice that overexpress IGF-I or II display specific alterations in mammary gland development such as an inhibition of mammary cell apoptosis following weaning and an increased incidence of mammary tumors. Thus, increased exposure to IGF-1 *in utero* may serve as a marker for the relationship between fetal growth and adult cancer susceptibility.

Although the effects of *in utero* exposure to dietary components have been inadequately examined, considerable evidence exists for their ability to modify IGF-1 concentrations and mammary cancer susceptibility postnatally. Postnatal caloric restriction decreases IGF-1 and decreases mammary tumor growth and metastases. Furthermore, postnatal soy phytochemicals combined with green tea synergistically inhibited mammary tumor growth and depressed serum IGF-1 levels in mice. Future studies are warranted to determine whether *in utero* exposure to dietary manipulations that modulate IGF-1 expression will influence subsequent breast cancer risk.

### Epigenetics, dietary modification, and breast cancer risk

Maternal nutritional status can also alter the epigenetic state of the fetal genome and imprint gene expression levels with lifelong consequences. Loss of imprinting is the silencing of active imprinted genes or the activation of silent imprinted genes, and is one of the most common epigenetic changes associated with the development of a wide variety of tumors. For example, loss of imprinting of IGF-2 has been associated with many different types of cancer, including mammary tumor development. H19, a tumor suppressor gene located directly downstream from IGF-2, is also genomically imprinted and is associated with various cancers. Furthermore, the hereditary disorder Beckwith-Wiedemann syndrome, which predisposes to cancer and causes prenatal overgrowth, involves alterations in IGF-2 and H19 imprinting. Several lines of evidence support the relationship between maternal nutrition and epigenetic changes in their offspring. First, a deficiency of amino acids results in marked reduction in genomic DNA methylation and aberrant expression of the normally silent paternal H19 allele in cultured mouse embryos. Second, uteroplacental insufficiency causes hypomethylation and increased histone acetylation in postnatal rat liver. Third, maternal supplementation of methyl donors and cofactors (folic acid, vitamin B-12, choline, and betaine) increases CpG methylation at the Avy locus of agouti mouse pups which causes a shift from a yellow to an agouti coat. The methylation patterns are retained into adulthood and are linked with a lower risk of cancer, diabetes, obesity, and prolonged life. Thus, epigenetic changes may provide a molecular mechanism for the impact of maternal nutrition or environmental chemical exposures on postnatal disease susceptibility and deserves future research.

Investigators may choose from the full range of preclinical approaches. The use of genetically engineered animal models, including transgenic or knockouts, such as those available through the mouse models of human cancer consortium (MMHCC; http://emice.nci.nih.gov/) are encouraged. Studies are encouraged that apply new high-throughput genomic, epigenomic, proteomic, and metabolomic technologies to determine how dietary and/or environmental chemical exposures *in utero* influence adult breast cancer susceptibility.

Illustrative examples for the development of R21 applications include, but are not limited to, the following examples: 1) utilization of transgenic and knockout mouse models of human mammary cancer to identify molecular sites of action of bioactive food components in cancer prevention; 2) examination of the role of moderate caloric restriction *in utero* on hormone concentrations (i.e., estrogen, insulin, ICF-1), and mammary cancer prevention; 3) evaluation of synergistic effects of exposure to bioactive food components *in utero* and subsequent mammary cancer risk; 4) evaluation of IGF-2, H19, and other imprinted genes after exposure to bioactive food components *in utero* and subsequent mammary cancer risk; 5) examination of the role of *in utero* exposures to environmental agents, such as mycotoxins, heterocylic amines, bisphenol A, pththlates, and other agents with endocrine-like agonist or antagonist activity and subsequent mammary cancer risk; and 6) examination of the interaction of *in utero* exposures to bioactive food components (e.g., phytoestrogens) and exposures to environmental agents in the etiology of breast cancer later in life.

This FOA will use the NIH Exploratory/Developmental Research Grant (R21) award mechanism. As an applicant, you will be solely responsible for planning, directing, and executing the proposed project.

This FOA uses just-in-time concepts. It also uses the modular budget formats: See the Modular Applications and Awards section of the NIH Grants Policy Statement. Specifically, if you are submitting an application with direct costs in each year of $250,000 or less (excluding consortium Facilities and Administrative [F&A] costs), use the PHS398 Modular Budget component provided in the SF424 (R&R) Application Package and SF424 (R&R) Application Guide (see specifically Section 5.4, Modular Budget Component, of the Application Guide).

Exploratory/developmental grant support is for new projects only; competing renewal (formerly competing continuation) applications will not be accepted. Up to two resubmissions (formerly revisions/amendments) of a previously reviewed exploratory/developmental grant application may be submitted. See NOT-OD-03-041, which was published in the NIH Guide on 7 May 2003.

Applicants must download the SF424 (R&R) application forms and SF424 (R&R) Application Guide for this FOA through Grants.gov/Apply.

Note: Only the forms package directly attached to a specific FOA can be used. You will not be able to use any other SF424 (R&R) forms (e.g., sample forms, forms from another FOA), although some of the Attachment files may be useable for more than one FOA.

For further assistance, contact GrantsInfo; 301-435-0714 (telecommunications for the hearing impaired: TTY 301-451-0088) or by e-mail: GrantsInfo@nih.gov.

Prepare all applications using the SF424 (R&R) application forms and in accordance with the SF424 (R&R) Application Guide (MS Word or PDF).

The SF424 (R&R) Application Guide is critical to submitting a complete and accurate application to NIH. There are fields within the SF424 (R&R) application components that, although not marked as mandatory, are required by NIH (e.g., the Credential log-in field of the Research & Related Senior/Key Person Profile component must contain the PD/PI’s assigned eRA Commons User ID). Agency-specific instructions for such fields are clearly identified in the Application Guide. For additional information, see Tips and Tools for Navigating Electronic Submission on the front page of Electronic Submission of Grant Applications.

The application submission dates are available at http://grants.nih.gov/grants/funding/submissionschedule.htm. The complete version of this PA is available at http://grants.nih.gov/grants/guide/pa-files/PA-06-277.html.

Contacts: Cindy D. Davis, National Cancer Institute (NCI), Division of Cancer Prevention, National Cancer Institute, 6130 Executive Boulevard, EPN Room 3159, MSC 7328 Bethesda, MD 20892-7328 USA (for U.S. Postal Service express or regular mail), Rockville, MD 20852 USA (for express/courier delivery), 301-594-9692, fax: 301-480-3925, e-mail: davisci@mail.nih.gov; Mary Frances Picciano, NIH Office of Dietary Supplements (ODS), Office of Dietary Supplements, Office of the Director, National Institutes of Health, 6100 Executive Boulevard, Room 3B01, MSC 7517 Bethesda, MD 20892-7517 USA (for U.S. Postal Service express or regular mail) Rockville, MD 20852 USA (for express/courier delivery) 301-435-3608, e-mail: piccianM@mail.nih.gov; Jerry Heindel, National Institute of Environmental Health Sciences (NIEHS), Cellular, Organs and Systems Pathobiology Branch, Division of Extramural Research and Training, PO Box 12233 RTP, NC, 27709 USA, 79 T.W. Alexander Drive, 4401 Building (for express/courier service) 919-541-0781, fax: 919-541-5064, e-mail: heindelj@niehs.nih.gov.

Reference PA-06-277

## NIH Pathway to Independence (PI) Award (K99/R00)

One of the most challenging transitions in any research career is the transition from postdoctoral trainee to independent scientist. Recent reports from the National Research Council of the National Academies of Science (Bridges to Independence: Fostering the Independence of New Investigators in Biomedical Research http://books.nap.edu/catalog/11249.html, and Advancing the Nation’s Health Needs: NIH Research Training Program http://www.nap.edu/booksearch.php?term=%22nrc+analysis%22&isbn=0309094275) have highlighted the need for enhanced efforts to foster the transition of postdoctoral scientists from mentored environments to independence.

The NIH data indicate that the average age of first-time (new) principal investigators obtaining R01 research funding from the NIH has risen to 42 years for Ph.D. degree holders and 44 years for M.D. and M.D./Ph.D. degree holders. This trend must be curtailed in order to capture the creativity and innovation of new independent investigators in their early career stages to address our nation’s biomedical, behavioral, and clinical research needs.

The primary goal of this pilot initiative is to facilitate receiving an R01 award earlier in a research career and to assist investigators in securing a stable research position during the critical transition stage of their career.

In addition to this initiative, NIH Institutes and Centers support a variety of mentored career development programs designed to foster the transition of new investigators to research independence. These programs span research career development opportunities for investigators who have made a commitment to focus on patient-oriented research through the Mentored Patient-Oriented Research Career Development Award (K23) http://grants.nih.gov/grants/guide/pa-files/PA-05-143.html to research career development opportunities for individuals with highly developed quantitative skills seeking to integrate their expertise in research relevant to the mission of NIH (K25) http://grants.nih.gov/grants/guide/pa-files/PA-06-087.html. Information describing all NIH Career Development Award programs can be found at http://grants.nih.gov/training/careerdevelop-mentawards.htm.

The NIH Pathway to Independence Award will provide up to 5 years of support consisting of two phases. The initial mentored phase will provide support for salary and research expenses for up to 2 years for the most promising and exceptionally talented new investigators who have no more than 5 years of postdoctoral research training experience at the time of initial application or subsequent resubmission(s). This initial phase of mentored support will allow the candidate time to complete research, publish results, and bridge to an independent research position. As part of the application, the candidate must propose a research project that will also be pursued as an independent investigator during the second phase of the award. The candidate and mentors together will be responsible for all aspects of the mentored career development and research program. An individual may submit an application from an extramural or intramural sponsoring institution/organization that has a rich and extensive research program in the area of interest as well as the faculty, facilities, and resources to support the proposed research endeavor. The individual must select an appropriate mentor with a track record of funded research related to the selected research topic and experience as a supervisor and mentor. The sponsoring institution must ensure that the candidate has the protected time needed to conduct the proposed research.

Following the mentored phase, the individual may request up to three years of support to transition, as an independent scientist, to an extramural sponsoring institution/organization to which the individual has been recruited. This support is to allow the individual to continue to work toward establishing his/her own independent research program and prepare an application for regular research grant support (R01). Support for the independent phase, however, is not automatic and is contingent upon being accepted by an extramural institution and the successful NIH programmatic review of the individual’s mentored phase of the award.

This funding opportunity will use the new combination K99/R00 funding mechanism. As an applicant, the candidate and his/her mentor are jointly responsible for planning, directing, and executing the proposed mentored phase of the research project.

This funding opportunity uses the just-in-time budget concepts. It also uses the nonmodular budget format described in the PHS 398 application instructions (see http://grants.nih.gov/grants/funding/phs398/phs398.html). The applicant should follow the instructions for budget information described in the PHS 398, Section III, providing only the total direct costs requested for each year and the entire proposed period of support and budget justification information.

The PHS 398 application instructions are available at http://grants.nih.gov/grants/funding/phs398/phs398.html in an interactive format. Applicants must use the currently approved version of the PHS 398. For further assistance contact Grants Info, 301-435-0714 (telecommunications for the hearing impaired: TTY 301-451-0088) or by e-mail: GrantsInfo@nih.gov.

Applications must be prepared using the most current PHS 398 research grant application instructions and forms. Applications must have a Dun & Bradstreet (D&B) Data Universal Numbering System (DUNS) number as the universal identifier when applying for federal grants or cooperative agreements. The D&B number can be obtained by calling 866-705-5711 or through the web site at http://www.dnb.com/us/. The D&B number should be entered on line 11 of the face page of the PHS 398 form.

The application submission dates for this PA are available at http://grants.nih.gov/grants/funding/sub-missionschedule.htm. The complete version of this PA is available at http://grants.nih.gov/grants/guide/pa-files/PA-06-133.

Contacts: Please see http://grants.nih.gov/grants/guide/contacts/pa-06-133_contacts.htm for this PA.

Reference: PA-06-133

## Research On Ethical Issues In Human Subjects Research (R03)

The common characteristic of the small grant is provision of limited funding for a short period of time. Examples of the types of projects that ICs support with the R03 include pilot or feasibility studies; secondary analysis of existing data; and small, self-contained research projects

This Funding Opportunity Announcement (FOA) issued by the National Institutes of Health (NIH) solicits Small Grant (R03) applications addressing ethical issues that accompany the conduct of research involving human subjects.

The purpose of this funding opportunity announcement is to solicit research addressing the ethical challenges of human subjects research in order to optimize the protection of human subjects and enhance the ethical conduct of human subjects research.

Recent developments in biomedical and behavioral research, which include the rapid growth of new interventions and technologies, increasing involvement of foreign populations in human subjects research, and concerns about financial conflicts of interest among researchers, challenge investigators’ abilities to interpret and apply the regulations. Other situations (e.g., research with vulnerable populations, research on stigmatizing diseases or conditions) may present difficulties for identifying strategies, procedures, and/or techniques that will enhance/ensure the ethical involvement of human subjects in research. Thus, research on ethical issues in human subjects research is necessary to enhance interpretation and application of ethical principles and regulatory requirements.

The research design for studies on ethical issues in human subjects research should be appropriate to the nature of the project(s) proposed and the disciplines involved. Given the conceptual and methodological complexity of many of these research questions, interdisciplinary and collaborative projects are encouraged, particularly those involving clinical researchers, ethicists, and behavioral/social scientists.

In conducting research on ethical issues in human subjects research, different conceptual frameworks for ethics (e.g., principlism, deontology, utilitarianism, rights, ethics of care) exist and may provide presuppositions and theoretical foundations from which bioethical questions can be formulated and tested. The questions and strategies for testing these issues must be consistent with existing federal requirements. Currently, research supported by the Department of Health and Human Services (DHHS; which includes NIH) follows the Code of Federal Regulations—Protection of Human Subjects (45 CFR Part 46). For research conducted internationally, alternative guidelines that describe protections equivalent to those required by 45 CFR 46 may be used (http://www.hhs.gov/ohrp/humansubjects/assurance/filasur.htm), such as those developed by the World Health Organization, the Council for International Organizations of Medical Sciences, and other internationally recognized groups.

This FOA seeks Small Grant (R03) applications for empirical or conceptual research that address the ethical challenges of research involving human subjects with the goal of optimizing protections. See http://grants.nih.gov/grants/funding/r03.htm for a description of the NIH Small Grant Program. The NIH is also issuing FOAs on the same topics using two other grant mechanisms: The R01 will support empirical research; the R21 will support conceptual as well as empirical research.

See http://grants.nih.gov/grants/guide/contacts/pa-06-367_368_369_differences.doc for an explanation of R01, R21, and R03 mechanisms.

Examples of the types of topics that would be appropriate for applications submitted under these announcements include, but are not limited to, the following:

1. Assessing risks in human subjects research: *a*) Assess how perceptions of risk may differ among investigators, IRB members, and potential subjects and their families, groups, and communities. Examine how features of the research, context of the research, or characteristics of research subjects (e.g., age, health status and stage of disease including those near the end of life, ethnic/cultural background, cognitive capacity, emotional or mental state, social status, sex, incarceration, enthusiasm/optimistic expectation about research) may alter risk perception. Identify and evaluate strategies to respond to differing perceptions of risks by stakeholders. *b*) Assess the severity and frequency of social, psychological, and/or economic harms (e.g., stigma, discrimination, personal and familial distress, depression, breaches of confidentiality, loss of insurance coverage, loss of employment, loss of housing, loss of benefits, domestic violence, incarceration) that may be associated with participation in or withdrawal from research. Develop and test methods to evaluate prospectively and minimize psychosocial risks in research. For example, formative research in a community may identify social or cultural issues or concerns before the conduct of research. Researchers may compare different methods to reduce risk, such as, for example, modifications to study design to reduce risks, or provision of additional services to research subjects. *c*) Develop and test models of prospective risk assessment in human subjects research, including, but not limited to, risks of new technologies. Risk assessment can be particularly challenging in the case of early human trials of new technologies. How can preclinical data best be used to assess the risk of new technologies used in first human trials? How can estimates of risk be translated from animal and *in vitro* studies to clinical trials in human populations? How can the need to advance new technologies into clinical application be balanced with caution about exposing research subjects to unknown risks, and specifically what scientific and ethical criteria are relevant to making these determinations? How can an assessment of the risks and benefits of research take into account the severity of disease and the urgency of need for new treatments as well as the possibility of therapeutic misconception? *d*) Develop models for design of clinical trials of new technologies. What study designs are most appropriate to allow consideration of safety issues in the first human trials? How can information from clinical trials of related interventions be used in designing new trials and in assessing safety data? *e*) Assess risks to privacy and confidentiality. Specific topics might include (but are not limited to): models for assessing confidentiality risks in research, including interventional research, social and behavioral research, and research with data and specimens; research to assess the use and understanding of certificates of confidentiality and the role they play in IRB assessments of risks to subjects.
2. Issues in informed consent: *a*) Identify and evaluate strategies, procedures, or techniques for improving comprehension and voluntariness in the process of informed consent for research. This could include evaluation of communication processes that take place before, during, or after the research, and/or measurement of comprehension, willingness to participate or continue participation, assent, and attitudes toward research. Examine how different aspects of the informed consent process affect these outcomes, including such parameters as: mode of presentation (e.g. oral, written, graphic, video); readability, complexity, and format of the language used; presentation of positive or negative aspects of participation in research, relative emphasis on benefits or burdens of participation; characteristics of the subject, such as language preference, age, sex, health status, education, emotional or mental state, cognitive capacity, cultural or ethnic background, views about medical professionals, and personal motivations; contextual features or circumstances in which informed consent takes place, including characteristics of the research staff; presence of an interpreter, location of the research (e.g., research hospital, private office, home); involvement of family members; involvement of subject advocates. *b*) Evaluate different methods and identify best-practice strategies for consulting with communities in the United States and/or other countries regarding comprehension, willingness to participate, or willingness to continue with research at the individual, group, community, or population level. *c*) Assess stakeholder attitudes regarding re-contacting subjects to obtain informed consent for additional uses of their data or to invite participation in other studies; examine the effect of re-contact on comprehension and willingness to participate. *d*) Assess the extent to which prospective subjects’ decisions about joining research are voluntary. Explore models of decision making and the effect of situational and individual characteristics on the decision-making process. Evaluate investigators’ attitudes regarding participants’ decision-making process. Assess the effects of incentives such as monetary compensation, provision of medical care, or other benefits of research on decision making and perceptions of research by potential or actual participants or study communities. *e*) Assess the impact of communicating or not communicating individual test results, incidental findings, study progress, and/or study results on participants’ willingness to continue participating in research and/or on attitudes towards research. *f*) Assess the ethical and legal implications of various models to handle illegal behavior by participants enrolled in or being screened for a study. *g*) Knowledge or perception of conflict of interest on the part of the investigator(s) or institution: Assess the impact of disclosing varying degrees of financial conflicts of interest involving the principal investigator, members of oversight committees, sponsor, institution, etc., on subjects’ willingness to participate and/or continue with research, and/or subjects’ understanding of the research.
3. International research: *a*) Evaluate different methods and identify best-practice strategies for consulting with communities and stakeholders in host countries as well as in the United States during all phases of human subjects research (planning, execution, and dissemination of results). Identify and test models from participatory research that are appropriate in different settings. Community consultation might address issues such as the significance or priority of the research project in light of local public health priorities; choice of timing of consultation during the development of the research plan; assessment of community members’ likely comprehension of the research; community perspectives on the design of the research; the willingness of community members to join or continue participation in research; and plans for feedback of information to communities during and after the research. *b*) Identify and evaluate strategies for investigators and sponsors of research to build trust and collaborative partnerships with host country communities, investigators and health care delivery systems. *c*) Evaluate how foreign human subjects protection systems are applied to U.S.-funded international research. Evaluate the extent to which such protections could satisfy U.S. regulatory requirements for equivalent protections. Evaluate experiences of investigators dealing with foreign regulatory systems and develop models for dealing with multiple human subjects protections systems and standards. *d*) Identify and evaluate strategies to protect research subjects in countries with different political and cultural environments and different attitudes toward personal autonomy. *e*) Develop methods to assess vulnerability, including economic and social vulnerability, in resource-limited settings and evaluate remedies to enhance human subjects protections of vulnerable populations or groups. *f*) Address the ethical and practical aspects of providing medical care in the context of human subjects research in low-resource settings, particularly in the international setting. Assess stakeholders’ perceptions of requirements or requests to provide ancillary care during the conduct of human subjects research. *g*) Identify and evaluate strategies to build health care and/or human subjects research capacity; assess durability of capacity building strategies; recommend best practices.
4. Study design in clinical trials and its relationship to medical care: *a*) Identify the ethics implications of specific scientific parameters in clinical trial design, such as the choice of appropriate control arm(s) in clinical trials of biomedical and/or behavioral interventions. Develop and/or evaluate strategies to address study design issues that pose particular ethical and scientific challenges. *b*) Evaluate how different study designs might entail different risks or benefits to subjects or to society at large in terms of social value of the research, including issues such as social acceptability, protection of the welfare of subjects in the trial, scientific rigor, relevance, feasibility, and timeliness. *c*) Evaluate the scientific and ethical implications of different types of controlled trials, including consideration of choice of flexible versus controlled conditions, physician or patient preferences in medical care, and strategies for handling scientific and medical disputes about usual or standard care. These issues can arise in studies that include biomedical interventions, behavioral interventions, or both. *d*) Evaluate the scientific and ethical acceptability of placebo-controlled trials in different scenarios, the societal and individual benefit from different trial designs and criteria to guide the conduct of trials using placebo or active controls.
5. Research oversight: IRBs, DSMBs, and COI committees: *a*) Identify and evaluate strategies to improve the oversight of protections for human subjects by IRBs. Examples might include: *i*) assessment of different models of IRB review, such as cooperative review arrangements for multisite or complex research protocols; collaborative IRB arrangements for multi-site studies or complex research projects; collaborative IRB arrangements in international studies; or division of labor among multiple IRBs reviewing the same research plan; *ii*) development and testing of models for effective communication, delegation of responsibilities, and joint decision making of multiple institutional review boards in international research; *iii*) assessment of stakeholder attitudes toward different IRB models including views of researchers, IRB members, institutional officials, community members, and research sponsors; *iv*) development of appropriate outcomes measures and quality indicators for the IRB review process for measurement of adequate protection of human subjects; development and testing of a framework for assessing IRB review quality; determination of when variability in IRB outcomes would be acceptable and when such variation would indicate inconsistent quality. *b*) Develop and test models or outcome measures for assessing the functioning of data and safety monitoring boards (DSMB). For example, develop and test models for effective communication, delegation of responsibilities, and joint decision making among multiple IRBs and DSMBs; compare and evaluate different methods and strategies for facilitating the submission and enhancing the interpretation of adverse event reports submitted to review bodies. Compare and evaluate different methods and strategies for identifying, reporting, and handling adverse events, severe adverse events, or unanticipated problems based on the perspectives of individual participants, institutions, DSMBs, and/or IRBs. *c*) Evaluate effectiveness of conflict of interest (COI) committees. For example, identify and evaluate strategies to address nonfinancial COI among research teams, sponsors, advisory boards, or other stakeholders. Assess the impact of perceived or real conflicts of interest among members of oversight committees on decision making about the acceptability of research protocols, interpretations of adverse events, and/or perceptions of “independence of review” by the research community.
6. Research with specimens and data: *a*) Assess the perspectives and attitudes of subjects about the use of specimens and/or data in research, including special cultural or religious beliefs and attitudes concerning the use of discarded clinical specimens. *b*) Assess how much subjects expect to know about how their specimens and/or data will be used in research. *c*) Develop novel approaches for obtaining informed consent for research use of specimens and data collected during the course of routine clinical care. *d*) Explore and develop ways of addressing ethical issues related to the return of individual research results to subjects, including studies of associated risks and harms. *e*) Explore and develop ways of addressing ethical issues related to intellectual property and ownership of specimens. *f*) Explore issues related to benefit sharing in the context of tissue research. Identify models and mechanisms that can be used to engage communities, sponsors, and researchers in benefit-sharing arrangements and assess the social and ethical acceptability and practicability of these arrangements. *g*) Identify and evaluate strategies for balancing the responsibility for protecting and/or minimizing disclosure of private information with the obligation to maximize the social value of research when identifiable data are collected via the Internet or other electronic method; or preserved for secondary analysis, e.g., a tissue or gene bank, data archive, or warehouse.
7. Dissemination of research findings: *a*) Explore when and how research results and incidental findings should be reported and disseminated to lay communities, including research subjects and the general public. *b*) Develop and evaluate methods for communicating research results and incidental findings with medical implications to research subjects and families. *c*) Develop and evaluate scientific and ethical parameters that should be considered when reporting generalized, aggregate research findings; develop and evaluate methods for communicating the evolving and sometimes conflicting nature of scientific evidence to lay audiences.

To assist you in identifying which NIH Institute/Center most closely matches your research topic, the following website provides additional information about Institute/Center-specific research interests that will be supported by this FOA: http://grants.nih.gov/grants/guide/contacts/pa-06-367_368_369_contacts.doc

This Funding Opportunity Announcement (FOA) invites applications for small research projects that can be carried out in a short period of time with limited resources. The applicant will be solely responsible for planning, directing, and executing the proposed project.

This FOA uses just-in-time concepts. It also uses the modular budget formats (see the Modular Applications and Awards section of the NIH Grants Policy Statement). All applications submitted in response to this FOA must use the modular budget format. Specifically, if you are submitting an application with direct costs in each year of $250,000 or less (excluding consortium Facilities and Administrative [F&A] costs), use the PHS398 Modular Budget component provided in the SF424 (R&R) Application Package and SF424 (R&R) Application Guide (see specifically Section 5.4, Modular Budget Component, of the Application Guide).

Competing renewal (formerly competing continuation) applications will not be accepted for the R03 grant mechanism. Small grant support may not be used for thesis or dissertation research. Up to two resubmissions (formerly “revisions/amendments”) of a previously reviewed small grant application may be submitted as defined in NIH Policy. See NOT-OD-05-046 http://grants.nih.gov/grants/guide/notice-files/NOT-OD-05-046.html

For specific information about the R03 program, see: http://grants.nih.gov/grants/funding/r03.htm.

Applicants must download the SF424 (R&R) application forms and SF424 (R&R) Application Guide for this FOA through Grants.gov/Apply.

Note: Only the forms package directly attached to a specific FOA can be used. You will not be able to use any other SF424 (R&R) forms (e.g., sample forms, forms from another FOA), although some of the “Attachment” files may be useable for more than one FOA.

For further assistance contact GrantsInfo, 301-435-0714 (telecommunications for the hearing impaired: TTY 301-451-0088) or by e-mail: GrantsInfo@nih.gov.

Prepare all applications using the SF424 (R&R) application forms and in accordance with the SF424 (R&R) Application Guide (MS Word or PDF).

The SF424 (R&R) Application Guide is critical to submitting a complete and accurate application to NIH. There are fields within the SF424 (R&R) application components that, although not marked as mandatory, are required by NIH (e.g., the Credential log-in field of the Research & Related Senior/Key Person Profile component must contain the PD/PI’s assigned eRA Commons User ID). Agency-specific instructions for such fields are clearly identified in the Application Guide. For additional information, see Tips and Tools for Navigating Electronic Submission” on the front page of Electronic Submission of Grant Applications.

The SF424 (R&R) application is comprised of data arranged in separate components. Some components are required, others are optional. The forms package associated with this FOA in Grants.gov/APPLY will include all applicable components, required and optional.

The application submission dates for this PA are available at http://grants.nih.gov/grants/funding/submissionschedule.htm. The complete version of this PA is available at http://grants/nih.gov/grants/guide/pa-files/PAR-06-367. Paper applications will not be accepted.

Contacts: The complete list of agency contacts is available at http://grants/nih.gov/grants/guide/pa-files/PAR-06-367.

Reference: PAR-06-367.

